# Up-to-Date Diagnostic CT Standards for Acute Appendicitis: Wall Thickness and Intraluminal Fluid Thickness

**DOI:** 10.7759/cureus.48154

**Published:** 2023-11-02

**Authors:** Mohammad Wazzan, Ahmed Abduljabbar, Huda Khizindar, Aghnar Alzahrani, Renad M Aljohani, Rana Nahas, Rahf Aman, Shouq Tawfiq, Arwa Aldajani

**Affiliations:** 1 Department of Radiology, King Abdulaziz University Faculty of Medicine, Jeddah, SAU; 2 Department of Medicine and Surgery, Albaha University, Baha, SAU; 3 Department of Medicine, King Saud Bin Abdulaziz University for Health Sciences College of Medicine, Riyadh, SAU; 4 Department of Medicine, Faculty of Medicine, Ibn Sina National College, Jeddah, SAU; 5 Department of Medicine and Surgery, Batterjee Medical College, Jeddah, SAU; 6 Department of Medicine and Surgery, King Faisal University, Jeddah, SAU; 7 Department of Medicine and Surgery, Alfaisal University College of Medicine, Riyadh, SAU

**Keywords:** diagnostic accuracy, sensitivity and specificity, ct scan, lumen thickness, wall thickness, acute appendicitis

## Abstract

Acute appendicitis is a prevalent condition that requires accurate and timely diagnosis and management to avoid potential complications. Classically, the diagnosis of appendicitis is made using the appendicular outer-to-outer wall diameter. In this study, we examined the sensitivity and specificity of computed tomography (CT) scans for diagnosing acute appendicitis using wall thickness and lumen thickness rather than diameter.

This study included data from 350 patients who presented to the emergency department with clinically suspected acute appendicitis. All patients underwent a CT scan, and 62 radiologically positive patients underwent surgery. A radiological diagnosis was made using the conventional outer-to-outer wall diameter with a cut-off of 6 mm for a positive diagnosis. These 62 positive CT scans were reviewed and compared with surgical results.

The study showed that a threshold of 2.25 mm for appendicular lumen thickness is an excellent diagnostic tool for acute appendicitis, demonstrating a high sensitivity of 96.4% and a lower specificity of 67%. In contrast, 1.6 mm wall thickness indicates acute appendicitis, with 81.8% sensitivity and 84% specificity. However, the wall thickness remains inferior to the conventionally used measurement of 6.75 mm for appendicular diameter, with a sensitivity of 87.5% and a specificity of 100%.

## Introduction

Appendicitis is one of the most common causes of acute abdomen in adults and has a lifetime risk of 6%-7% [1]. It is defined as inflammation of the vermiform appendix, an acute condition that presents within 24 hours [2].

Multiple imaging modalities, including abdominal computed tomography (CT), ultrasonography, and magnetic resonance imaging (MRI), aid in the clinical diagnosis of appendicitis [2]. Recently, CT scans with high accuracy have been widely used in acute settings [2]. Moreover, CT scans provide high-resolution images that better evaluate potential complications, eliminate differential diagnoses, and limit unnecessary surgical explorations [3,4]. Consequently, the number of unnecessary appendectomies has significantly decreased owing to the use of CT scans. Further, the perforation rate decreases in parallel.

The primary criteria for a CT scan to diagnose acute appendicitis are appendicular swelling, defined as an appendicular diameter greater than 6 mm, and periappendiceal inflammatory alterations. Nevertheless, normal appendices with a diameter >6 mm are frequently encountered in daily practice [5]. In contrast, we occasionally encounter patients with appendicitis who do not exhibit significant periappendiceal inflammation [5].

There are no reports describing the differences observed in CT parameters between a normal appendix of more than 6 mm and an inflamed appendix that does not exhibit periappendicular fat stranding. Differentiation between these entities may be challenging, especially without a clear clinical presentation, such as in cases of vague abdominal pain.

In this study, we evaluated the accuracy of measuring appendicular wall thickness and intraluminal fluid thickness as supportive methods for CT scans in diagnosing acute appendicitis.

## Materials and methods

Study design

This study is a retrospective cross-sectional study conducted at King Abdulaziz University Hospital, Jeddah, Saudi Arabia. The study analyzed data from 350 CT scans of patients who presented to the emergency department with clinically suspected acute appendicitis and correlated it with the final diagnosis by reviewing the emergency room notes and progress notes in the surgical notes. Sixty-two patients were positive for CT scan criteria and underwent surgery. The CT scans of these 62 patients were reviewed for lumen thickness and wall thickness and compared with the surgical results of the patients.

Data collection

Emergency room notes, surgical notes, and progress notes were obtained from the hospital information system and thoroughly reviewed. All data were secured by the principal author.

Inclusion criteria

The study included all positive CT scans for acute appendicitis that were confirmed positive after surgery. A radiological diagnosis of acute appendicitis was made using the conventional outer-to-outer wall diameter with a cut-off of 6 mm for a positive diagnosis. Patients who underwent appendectomy, percutaneous drainage, or received antibiotics were categorized as confirmed positives after confirmation from the surgeon's operation notes.

Exclusion criteria

All CT scans negative for acute appendicitis have been excluded.

Reference standard

Patients who underwent appendectomy and the surgeon’s operation note documented a normal or non-inflamed appendix or had alternate diagnoses and managed accordingly and did not present with similar complaints to our hospital within our research study time frame were categorized as confirmed negative.

Statistical analysis

IBM SPSS software, version 25 (IBM Corp., Armonk, NY), was used for statistical analysis. Descriptive analysis was performed using the quantitative variables’ mean and standard deviation (SD). For qualitative variables, we calculated the frequencies and percentages. We calculated accuracy measures, including sensitivity, specificity, and area under the receiver operating characteristic (ROC) curve (AUC). Statistical significance was set at a confidence interval (CI) of 95% and a margin of error of < 0.05.

Data collection

The measurements of the appendix, including the diameter, wall thickness, and lumen thickness, were obtained on the axial images of the CT scan. It was obtained by a senior radiology resident and reviewed by a senior radiologist.

Ethical approval

Per institutional policies and regulations, this research study did not require Institutional Review Board (IRB) approval due to the nature of the data and methods used.

## Results

The study analyzed data from 350 CT scans; 62 were positive by CT scans and confirmed positive after appendectomy. Our findings revealed that males accounted for 57.1% (32) of the positive scans, and females accounted for 42.9% (24). Additionally, patients with positive scans were more likely to have appendicoliths, fat stranding, and free fluids. These features are indicators of inflammation, infection, and appendiceal obstruction. In contrast, patients with negative scans had an equal distribution of males and females, were less likely to have any of the abovementioned features, and were likely to have intraluminal air. The mean age of the patients was 33 years. The mean appendiceal length was 7 cm, the mean diameter was 8.8 mm, the mean lumen thickness was 9.5 mm, and the mean wall thickness was 2.3 mm (Tables 1, 2).

**Table 1:** Descriptive analysis of the qualitative study variables This table presents the descriptive analysis of the study variables for 62 CT examinations.

Variable		Confirmed positive		Confirmed negative	
Sex	Male	32	57.1%	3	50.0%
	Female	24	42.9%	3	50.0%
Intraluminal air	Positive	13	23.2%	3	50.0%
	Negative	43	76.8%	3	50.0%
Appendicolith	Positive	14	25.0%	0	0.0%
	Negative	42	75.0%	6	100.0%
Fat stranding	Positive	56	100.0	0	0.0
	Negative	0	0.0	6	100.0
Free fluid	Positive	24	42.9%	0	0.0%
	Negative	32	57.1%	6	100%
Abscess/Phlegmon	Positive	3	5.4%	0	0.0%
	Negative	53	94.6%	6	100%

**Table 2:** Descriptive analysis of the quantitative study variables

Variable	Mean±SD	Minimum–Maximum
Age (year)	33.98±16.8	9–83
Length (cm)	7.0±1.7	3–13
Diameter (mm)	9.7±2.9	5.2–19
Lumen thickness (mm)	5.2±2.0	1.5–10
Wall thickness (mm)	2.3±0.80	1–4

These measurements help confidently diagnose acute appendicitis, considering that an enlarged appendix with thickened walls reflects a state of inflammation and infection.

Applying the 1.6 mm wall thickness measurement alone to diagnose acute appendicitis resulted in 81.8% sensitivity and 84% specificity. When utilizing a luminal thickness of 2.25 mm to diagnose acute appendicitis, the highest sensitivity was 96.4%, and the specificity was 67% (Table 3).

**Table 3:** Accuracy of wall thickness, lumen thickness, and appendix diameter in CT scans for the diagnosis of acute appendicitis AUC: area under the receiver operating characteristic curve; CI: confidence interval

Variable	Size (mm)	Sensitivity	Specificity	AUC	95% CI	
					Lower	Upper
Wall thickness	1.6	81.8%	84%	0.883	0.774	0.992
Lumen thickness	2.25	96%	67%	0.915	0.811	1.00
Appendix diameter	6.75	87.5%	100%	0.887	0.805	0.969

However, other studies have reported lower sensitivity and specificity. For example, Alshebromi et al. (2019) reported a specificity of only 16.7% [1]. A more recent study by Eurboonyanun et al. (2021) reported a sensitivity of 80.7% and a specificity of 86.7% for non-enhanced CT scans [4], which are lower than those of other studies that used enhanced contrast CT. These differences may be due to variations in the study population, imaging protocols, or interpretation criteria [6].

## Discussion

Appendicitis is a medical condition characterized by appendiceal inflammation. It most commonly presents as an acute condition that manifests suddenly and severely, often within 24 hours of onset [7]. The classic symptoms of appendicitis include vague periumbilical pain, anorexia, nausea, vomiting, migration of pain to the right lower quadrant, and low-grade fever [8].

All patients suspected of having acute appendicitis should undergo laboratory testing, including total white blood cell count, percentage of neutrophils, and measurement of C-reactive protein concentration, before proceeding to further diagnostic steps [9].

The mean age of the participants was 34 years. The appendix had a mean length of 7 cm and a mean diameter of 8.8 mm. The mean lumen thickness of the appendix was 9.5 mm, and the mean wall thickness of the appendix was 2.3 mm.

Among the patients with surgically proven positivity, males accounted for 57.1% (32) of the positive scans, and females accounted for 42.9% (24). In addition, positive cases were less likely to have intraluminal air; however, they demonstrated appendicoliths, fat stranding, and free fluids. These features can indicate inflammation, infection, or obstruction of the appendix. In contrast, negative cases had an equal distribution between males and females, demonstrated intraluminal air, and were less likely to have any of the features above.

Appendicular diameter is commonly used in daily clinical practice for diagnosing acute appendicitis on CT. When we evaluated the accuracy of this method, we found that the 6.75 mm appendicular diameter had a sensitivity of 87.5% and a specificity of 100%. This result is compatible with prior findings in the literature, suggesting that CT is a highly accurate method for diagnosing acute appendicitis. Eng et al. reported sensitivity and specificity of 94.9% and 92.4%, respectively [3]. Yun et al. reported a sensitivity of 96.40% and a specificity of 92.17% for standard-dose CT scans [10]. Doan et al. reported a higher sensitivity (97.4%) and accuracy (96.6%) [2].

However, other studies have reported conflicting results with lower sensitivity and specificity. Alshebromi et al. reported a specificity of only 16.7% [1]. Eurboonyanun et al. reported a sensitivity of 80.7% and a specificity of 86.7% for non-enhanced CT, which is lower than that of other studies that used enhanced contrast CT [4]. These differences may be due to variations in the study population, imaging protocols, or interpretation criteria [10].

Our proposed method suggests the application of lumen thickness and luminal thickness measurements to diagnose acute appendicitis. The results showed that 2.25 mm lumen thickness had a high sensitivity (96%) but a low specificity (67%), with an area under the ROC curve of 0.915. However, when applying 1.6 mm wall thickness, it showed a lower sensitivity of 81.8% but a higher specificity of 84%.

Overall, these results suggest that appendicular lumen thickness and wall thickness measurements on CT are important predictors of the likelihood of positive CT results in acute appendicitis. An increase in the lumen thickness was associated with a significantly higher likelihood of a positive CT result, which may aid in diagnosing acute appendicitis.

One limitation of this study is the relatively small sample size, which may limit the generalizability of the findings. Therefore, the results of this study should be interpreted with caution. Further validation studies with larger sample sizes are required to confirm our findings.

## Conclusions

The study included 62 confirmed positive CT scans. The appendicular lumen thickness of 2.25 mm is an excellent diagnostic tool for acute appendicitis, demonstrating a high sensitivity of 96.4% but a lower specificity of 67%. On the other hand, 1.6 mm wall thickness has 81.8% sensitivity and 84% specificity, which is inferior to the conventional appendicular thickness, yielding a sensitivity of 87.5% and a specificity of 100%.
